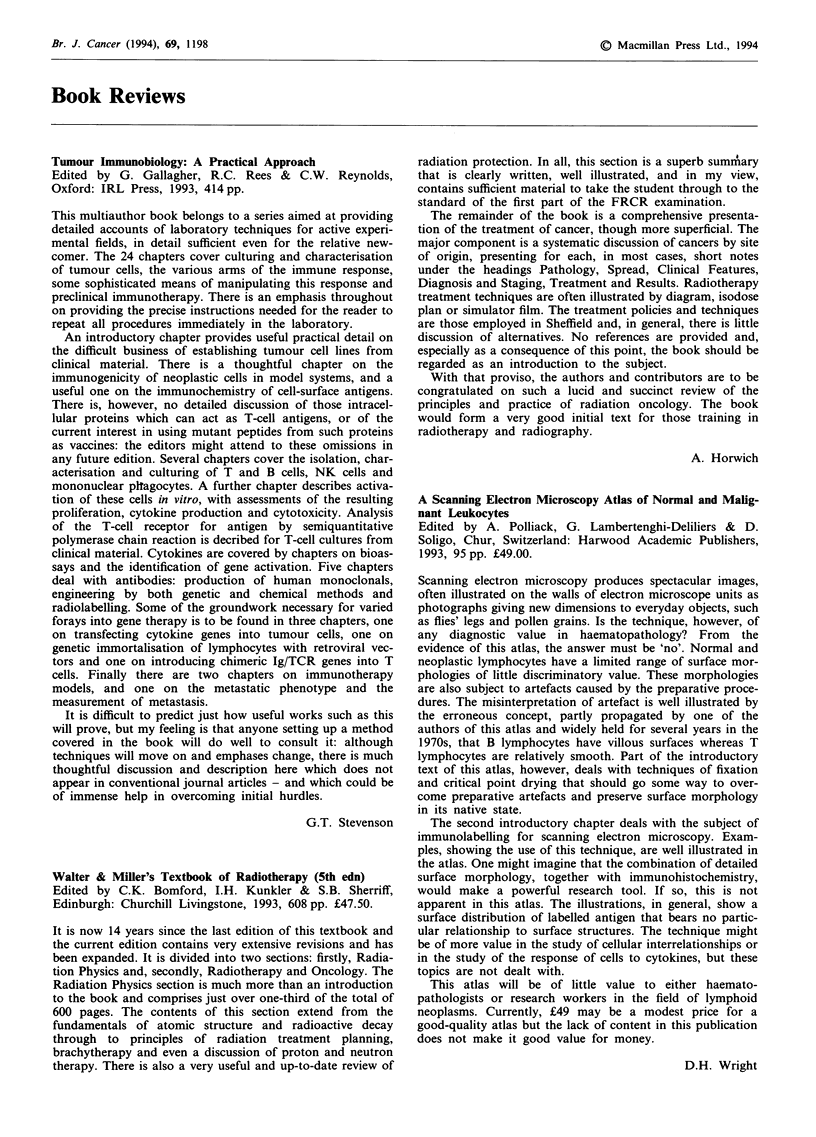# A Scanning Electron Microscopy Atlas of Normal and Malignant Leukocytes

**Published:** 1994-06

**Authors:** D.H. Wright


**A Scanning Electron Microscopy Atlas of Normal and Malignant Leukocytes**
Edited by A. Polliack, G. Lambertenghi-Deliliers & D. Soligo, Chur, Switzerland: Harwood Academic Publishers, 1993, 95 pp. £49.00.

Scanning electron microscopy produces spectacular images, often illustrated on the walls of electron microscope units as photographs giving new dimensions to everyday objects, such as flies' legs and pollen grains. Is the technique, however, of any diagnostic value in haematopathology? From the evidence of this atlas, the answer must be 'no'. Normal and neoplastic lymphocytes have a limited range of surface morphologies of little discriminatory value. These morphologies are also subject to artefacts caused by the preparative procedures. The misinterpretation of artefact is well illustrated by the erroneous concept, partly propagated by one of the authors of this atlas and widely held for several years in the 1970s, that B lymphocytes have villous surfaces whereas T lymphocytes are relatively smooth. Part of the introductory text of this atlas, however, deals with techniques of fixation and critical point drying that should go some way to overcome preparative artefacts and preserve surface morphology in its native state.

The second introductory chapter deals with the subject of immunolabelling for scanning electron microscopy. Examples, showing the use of this technique, are well illustrated in the atlas. One might imagine that the combination of detailed surface morphology, together with immunohistochemistry, would make a powerful research tool. If so, this is not apparent in this atlas. The illustrations, in general, show a surface distribution of labelled antigen that bears no particular relationship to surface structures. The technique might be of more value in the study of cellular interrelationships or in the study of the response of cells to cytokines, but these topics are not dealt with.

This atlas will be of little value to either haematopathologists or research workers in the field of lymphoid neoplasms. Currently, £49 may be a modest price for a good-quality atlas but the lack of content in this publication does not make it good value for money.

D.H. Wright